# Adverse events related to central venous catheters (CVC) and the influence of CVC characteristics on peripheral blood hematopoietic progenitor cell collection in children

**DOI:** 10.3389/fped.2023.1131905

**Published:** 2023-04-21

**Authors:** Josune Zubicaray, Sofía Martin-Consuegra, Monserrat Nieto, Gustavo Albi, June Iriondo, Elena Sebastian, Eva Gálvez, Blanca Molina, Marta González-Vicent, Jesus Gonzalez de Pablo, Ana Castillo, Manuel Ramírez, Luis Madero, Miguel Angel Díaz, Julián Sevilla

**Affiliations:** ^1^ Hematology and Hemotherapy Unit, Fundación para la Investigación Biomédica Hospital Infantil Universitario Niño Jesús, Madrid, Spain; ^2^ Pediatric Intensive Care Unit, Fundación para la Investigación Biomédica Hospital Infantil Universitario Niño Jesús, Madrid, Spain; ^3^ Radiology Department, Fundación para la Investigación Biomédica Hospital Infantil Universitario Niño Jesús, Madrid, Spain; ^4^ Hematopoietic Transplant Unit, Fundación para la Investigación Biomédica Hospital Infantil Universitario Niño Jesús, Madrid, Spain; ^5^ Hematology and Oncology Laboratory, Fundación para la Investigación Biomédica Hospital Infantil Universitario Niño Jesús, Madrid, Spain

**Keywords:** pediatric donors, CVC children, CVC adverse events, CVC apheresis, collection efficiency

## Abstract

**Introduction:**

The use of peripheral blood progenitor cells (PBPCs) as a source for hematopoietic stem cell transplantation (HSCT) in pediatric healthy donors is still under debate. The risk of a central venous catheter (CVC) placement and catheter-related complications continue to be the main arguments to discourage its use.

**Methods:**

we present a retrospective analysis of 140 PBPC collections in pediatric patients and donors, describing adverse events (AE) related to CVCs as well as the influence of catheterrelated variables on the efficiency of the leukapheresis.

**Results:**

14 CVC-related AEs were recorded (10%). The most common was fever in 5 patients, 4 of which had a catheter-related bacteriemia. Thrombotic events were only observed in 3 patients with active malignancy. A healthy donor presented a moderate bleeding after catheter withdrawal that resolved with local measures, and none of the rest presented any AE. Regarding variables related to the development of AEs, the subject group (patient or donor) was the only one significantly associated (*p* < 0.0001). Of interest, efficiency was also related to catheter location, being worse in those located in the femoral vein than in into the jugular or the subclavian veins (*p* < 0.05). In a multivariate analysis, the only variable significantly associated was catheter size (beta 0.238, *p* < 0.01).

**Discussion:**

Placing a CVC for PBPC collection in pediatric subjects is overall safe; CVC-related complications in pediatric healthy donors are very rare. Furthermore, we should try to place catheters of the largest caliber possible, since the efficiency of the collection is related to this variable.

## Introduction

Nowadays, autologous hematopoietic stem cell transplantation is almost exclusively performed by peripheral blood progenitor cells (PBPCs) collected after mobilization. This is also valid for gene therapy protocols using hematopoietic progenitor cells as the target cell for transduction in monogenic diseases (immunodeficiencies, sickle cell disease….). In adults, although the results are not conclusive regarding which procedure (bone marrow harvest or PBPC apheresis) is safer for stem cell donation, PBPC is the stem cell source most frequently used for hematopoietic transplantation in all series (1–3). However, the use of pediatric donors for PBPC collections is still under debate (1–5).The risk of catheter placement and catheter-related complications continue to be one of the main arguments used by critics of this approach to discourage its use (1, 4).

Although the adverse events related to central venous lines are not infrequent in pediatric patients, they have mainly been described in patients with several prothrombotic conditions at the time of the events or at high risk of infection (6–8). For instance, intensive care or oncology patients. However, healthy children are not at such a high risk, and complications are extremely rare (4, 9).

Based on our own experience in this setting we have designed a retrospective study to analyze adverse events related with central venous catheters used for PBPC collection (10–12). As a secondary aim of this study, we have analyzed several variables related to the catheter and their influence on the yield of PBPCs and in the efficiency of the leukapheresis.

## Materials and methods

Medical records of all pediatric patients and healthy donors (up to 18 years of age) that underwent PBPC collections in our unit since January 2011 *via* double lumen central venous catheter (CVC) were retrospectively reviewed. Written informed consent for the procedures, and the use of the registered data for investigational purposes was obtained from each child or their legal representative. The study was approved by the Hospital Infantil Universitario Niño Jesús (HIUNJ) ethics committee.

The primary aim of this study is to determine the incidence of adverse events related to the CVC, and the secondary one is the influence of the catheter size on the results of the collection.

All CVC were placed by the interventional radiology or the critical care unit teams at our center using mild sedation and local anesthesia.

### Apheresis

PBPC collection was performed using continuous flow blood cell separator (COBE Spectra TM, v.6.1, by Caridian BCT Europe, Garching, Germany; or Spectra Optia MNC v.3.0. or CMNC, Terumo BCT, Lakewood, Colorado) on the fifth day of mobilization.

Priming of the blood cell separator was performed with packed red blood cells (PRBC) for patients with <20 kg of body weight (BW). Priming with PRBC was avoided for donors, where it was substituted by a 4% albumin solution. Acid citrate dextrose (ACD-A) at a ratio of 14:1 was used as anticoagulant, and a calcium gluconate solution was continuously infused to prevent hypocalcemia.

Vital signs were monitored before and during the procedure.

### Collection results

Collection results were analyzed based on the number of CD34^+^ cells collected per BW. Collection efficiency (CE) was calculated as follows: (CE1%): total CD34^+^ cells collected x 100/[(pre-apheresis + post-apheresis CD34 + cells/mcl/2) × processed volume mcl].

Platelet and hematocrit loss were calculated as the difference between their determination immediately before and after the apheresis procedure.

### Adverse events

All patients and guardians were asked to report AEs during the collection, and a nurse team with documented experience on these procedures in children, recorded every single adverse event found during the procedure in a standardized registry form (13).

Blood cultures and catheter tip cultures were performed in all children with fever, local infection or thrombosis.

Doppler ultrasound studies were performed in patients with clinical signs and/or symptoms of thrombosis or catheter dysfunction.

### Statistical analysis

Quantitative variables are presented as the median and the interquartile range (IQR), and qualitative variables are expressed in percentages (%) and/or frequencies. Pearson's *χ*^2^ test was used for the bivariate analysis of categorical variables. Student's t-test or Mann–Whitney *U* or Kruskall–Wallis tests were applied for quantitative variables. Variables that showed a statistically significant association in the univariate analysis were then evaluated by logistical regression in a multivariate analysis. Statistical analysis was performed using SPSS software, version 22.0. A *p*-value <0.05 was considered significant.

## Results

We have analyzed the results of 140 PBPC collections performed in our unit since January 2011 in 140 pediatric patients and donors (<18 years). The main children and leukapheresis demographic characteristics are shown in Table 1.

**Table 1:** Demographic characteristics of the series.

Children	Median	IQR^†^
Age (years)	5	2–10
Weight (kg)	18	13–37.75
Total blood volumen (ml)	1,440	1,034–2,604
Patients/Donors	91/49	
Leukapheresis	Median	IQR^†^
Duration of apheresis session (min)	257	230–287
Number of blood volumes processed	3.3	2.9–4
Flow rate (ml/min)	19.34	14.11–34.65
CD34^+^ cell count before apheresis	56	33–98
CD34^+^ cells collected (CD34^+^ cells ×10^6^/kg)	5.2	3.19–9.10
Collection efficiency	49.27	40.16–60.72
Software device:		
COBE Spectra/Spectra Optia CMN/Spectra Optia CMNC/NR^‡^	40/17/80/3	

^†^ IQR, interquartile range.

^‡^ NR, not recorded.

In 131 cases (93.6%) the CVC was placed between the first day of mobilization and apheresis. Only 6.4% catheter insertions (9 cases) were carried out before. Therefore, in most cases the CVC was placed for the PBPC collection. Of the total of 140 cases, in 63.6% of them the CVC was removed in the following 72 h to apheresis procedure. That means that, in 36.4% of them the CVC was maintained beyond 72 h in order to be used in patients for the subsequent chemotherapy cycle. In fact, in 14.3% of cases (20 cases) this CVC was preserved between 1 and two months to be used for new chemotherapy cycles. Among them, only those that were tunneled (7) continues for more than 2 months.

### Catheter characteristics

Catheter location and commercial brand are shown in Table 2. Most catheters were placed in the jugular vein (52.1%) and the most frequent ones in our series were of the Vygon® brand.

**Table 2:** Central venous catheter location and brand.

	*N*	%
**Placement**		
Jugular	73	52.1
Subclavian	13	9.3
Femoral	54	38.6
**CVC^†^ Brand**		
Unknown	34	24.3
MedCOMP	6	4.3
Arrow	2	1.4
Vygon	94	67.1
Cook	4	2.9

^†^ CVC, central venous catheter.

Vygon: Ecouen, France.

MedCOMP, Medical Components, Inc. Haleysville, Pennsylvania, USA.

Arrow International, Inc., Division of Teleflex Medical Inc.: Everett, Massachusetts, USA.

COOK Medical: Bloomington, Indiana, USA.

Catheter size and length was recorded for 126 and 120 children respectively. The median CVC size for all children was 7 French (Fr) (IQR: 5–8), with the smallest one of 3 Fr used in only one patient (Table 3). Younger patients (less than 5 years old) had a median catheter size of 5 Fr and 8 cm of length, whereas in the older ones (≥5 years old) these were 8 Fr and 15 cm. For those children with less than 11 kg of weight, the median size and length was 5 Fr and 8 cm, and for the ones weighing 20 kg or more it was 8 Fr and 15 cm, respectively. Not surprisingly, size and length were clearly related to the age and size of the patients (*p* < 0.0001) (Table 3).

**Table 3:** Size and length of the central venous catheter.

	Size (Fr)	Length (cm)	*p*
	Median (IQR^†^)	Median (IQR^†^)	
**Weight**			
≤10 kg	5 (4–5)	8 (8–11.75)	<0.0001
11–19 kg	5 (5–7)	8 (8–15)	
≥20 kg	8 (7–8)	15 (15–15)	
**Age**			
<5 year old	5 (5–5.5)	8 (8–15)	<0.0001
≥5 year old	8 (7–8)	15 (15–15)	

^†^ IQR, interquartile range.

### Adverse events

Only one patient developed hypotension during catheter placement and required packed red blood cell transfusion during the procedure. None of the donors experienced any complication during this procedure.

Overall, 14 adverse events were recorded related to the CVC in this series (10%) (Table 4). The most common event was fever in 5 patients (3.6%). Four patients with fever as the main clinical symptom were considered to have a central line associated blood stream infection (CLABSI) orbacteriemia, since blood and CVC tip cultures resulted positive to *coagulase-negative Staphylococcus* (2), and *Enterococcus faecalis* (2). In the other case, *Streptococcus mitis* was isolated in the blood culture but the catheter tip was negative, and therefore considered as contamination. We also found two patients (1.4%) who developed catheter-related local infection. Of note, three of the CLABSI were described in patients with femoral catheters. However, this was not statistically significant (*p* = 0.64). All infections resolved after CVC removal and systemic antibiotic treatment.

**Table 4:** Central venous catheter (CVC) related adverse events.

	*n*	%
Fever	5	3.6
Thrombosis	3	2.1
Bleeding	3	2.1
Local CVC infection	2	1.4
CVC displacement	1	0.7

Thrombotic events (3 patients) were only observed in patients with active malignancy. These included a metastatic CNS tumor, a rhabdoid tumor and a disseminated neuroblastoma, and all of them receiving chemotherapy.

In one patient, the CVC was removed due to displacement and it was relocated.

Only one of the adverse events recorded was presented in a healthy donor. He was a 13 year old male who developed moderate bleeding after removal of the CVC and was managed with a compressive bandage and oral antifibrinolytics. He had no previous history of bleeding, and standard hemostatic studies were performed without significant findings.

We also analyzed variables related to the development of AEs. Development of AEs due to the CVC was statistically related to the main group (patient or donor) (*p* < 0.0001). We could also found that CLABSI were related to the total time that the CVC was present. Median time for those cases that developed CLABSI was significantly longer (38 days; IQR: 13.5–58 days) than for those without infection (1 day; IQR: 1–11.75 days), *p* < 0.01. No statistically significant differences were found between other cohort baseline characteristics (age, sex, and diagnosis) and/or CVC specifications (location, length, size).

### PBPC collections

Median CD34^+^ cell count in peripheral blood before collection and median CD34^+^ cell collected are reported in Table 1.

In the multivariate analysis, the CD34^+^ cell count in peripheral blood preapheresis and the processed blood volume per kilogram of body weight were the only variables related to the CD34^+^ cells collected per kilogram of body weight (Table 5). Of interest, the flow rate during the leukapheresis procedure was also related to the amount of PBPC collected (*p* = 0.04) in the univariate study. Donor collections were also better than those performed to patients, as expected. The median number of CD34^+^ cells collected per kilogram of body weight in healthy donors was 6.08 × 10^6^ (IQR: 1.09–11.76), while for patients it was 4.7 × 10^6^ (IQR: 2.8–8.16 × 10^6^) (*p* = 0.018). However, as mentioned above, these correlations were not statistically significant after the multivariate analysis. Neither sex, age, catheter location, blood cell separator, nor software program used for collection were related to the CD34^+^ cells collected.

**Table 5:** Variables related to CD34 + cells collected per body weight.

Variable	β	β (range)	Standaryzed β	*p*
CD34^+^ cell count in PB preapheresis	0.06	0.052–0.068	0.828	<0.0001
Blood volume processed per kilogram body weight	0.015	0.004–0.027	0.145	0.011

### PBPC collection efficiency

Median collection efficiency for this series is close to 50% (Table 1). We found a linear correlation between efficiency and age (*p* < 0.01), weight (*p* < 0.001), catheter size (*p* < 0.01), and flow rate (*p* < 0.001).

Efficiency is also related to the catheter location, being worse in those located in the femoral vein (median 44.4%, IQR: 37.8–53.8) than in the ones placed into the jugular (median 56%, IQR: 41.9–63.3) or the subclavian veins (median 51.8%, IQR: 42.8–63.2) (*p* < 0.05). We believe that this difference might be related to the size of the catheters, since most of those located in the femoral vein (61.9%) had a smaller diameter ≤5 Fr, in comparison to ≥7 Fr in most of those located in the jugular vein (55.6%) or in the ones placed in the subclavian vein (75%) (*p* < 0.0001).

Moreover, efficiency is also related to the software used for collection. The procedures performed with the old device COBE Spectra had a median efficiency of 44% (IQR: 37.7–54.2), those using the MNC software of the Spectra Optia device showed a median of 71.1% (IQR: 56.6–78.8), and those performed with the Spectra Optia device with the CMNC program a median of 50% (IQR: 42.4–60.6) (*p* = 0.0001).

Collection efficiency was not related with the hematocrit or platelet loss.

When all these variables were included in a multivariate analysis, the only one that showed statistically significant association was catheter size (beta 0.238, *p* < 0.01). Those procedures performed with catheters with a size ≤5 Fr had a median efficiency of 45.6% (IQR: 38.7–57.8), vs. a 50.7% (IQR: 41–70.8) in those with a catheter size >5 Fr (*p* = 0.026).

## Discussion

The need for CVC placement for PBPC collection is considered to be one of the greatest sources for complications of the donation process and it is frequently discussed in different studies. This is even more relevant in the case of pediatric donations, where some teams even consider it as the main variable for choosing bone marrow donation over peripheral blood. For this reason, we decided to review the adverse events related to catheter placement and throughout the donation procedure, not only in pediatric healthy donors but also in patients. To our knowledge, this is the first study that aims to differentiate complications between these two populations, as well as to identify catheter-related variables that may influence their appearance and the final results of the collections.

We found that adverse events related to the CVC are infrequent in pediatric donors. In only one case, we had a moderate bleeding requiring treatment after catheter withdrawal. None of the other donors included in this study presented any adverse event related to the CVC. All the other adverse events were described in patients, most of them carrying the CVC for longer periods than the donors, who usually have the CVC for two or three days. We usually place the CVC the day before the collection and withdraw it some hours after the collection is completed. As previously stated, there is little information on adverse events in pediatric donors related to CVC (4). This extremely low incidence of adverse events related to CVC is also found in another study perfomed by the European Society Bone Marrow Transplantation (EBMT), in which they report only 1 case of pneumothorax with hydrothorax in a 5-year-old (4). However, since the complication was severe in that case, it is frequently used by critics of PBPC collection in children to discourage its use. In that prospective study, only one of 140 children as above mention presented a severe adverse event related to the CVC (0.7%). No other adverse event related to the CVC are described in that study, although the authors also describe minor complications of anesthesia after CVC placement: vomiting (2), decreased blood pressure (1), tachycardia (2), and bradycardia (1). None of them were described in our donors.

It is well recognized in pediatrics that CVCs are closely related to thrombosis and infection. Therefore, there is still a trend to relate the use of these devices to severe complications. However, as pointed out in our study, these complications are extremely rare in PBPC pediatric donors, and almost exclusively described in sick children.

Infections are the most frequent complications in our series, reaching a total of 5% if we group together fever originating from the CVC and local infections. They have only been described in patients, hence, in cases in which CVC manipulation was more frequent, and their duration was also longer. Frequent manipulations, blood draws, infusions, and the time that the CVC is being used are variables frequently related to these complications in other series (8).

Another interesting finding of our study is the relationship between the size of the CVC and the efficiency of the collection. There are several interesting reviews on pediatric apheresis with different recommendations on venous accesses and CVC insertion. Del Fante et al. summarize these methodological issues in a recent publication (14). The authors recommend to make a special reference to a good size catheter that guarantees an appropriate and stable blood flow during the harvest. However, there are no published studies that support that this statement could be related to better or more efficient collection, which on the other hand seems obvious. The size of CVCs reported in literature ranges from 7 to 12 Fr, but in the smaller children this team reports the use of 6 Fr catheters. In our study, the smallest one used was a 3 Fr one, although this was exceptional. We have several procedures performed through a 5 Fr central line without significant complications and good collections, but worse efficiencies as herein demonstrated. In general, we always try to place a catheter with the largest caliber possible, and that does not damage the vessel due to the increased risk of thrombosis. With the data presented here, it is obvious that the procedures performed with catheters with higher caliber (>5 Fr) have greater efficiency than the other ones. The reasons that can lead to this result can be different: less time to establish the interface, fewer stops during the collection, more stable flows, higher collection speeds, etc. We do not know which of all of them, or if the combination of some of them can play a role in the differences found, but the truth is that as we suspected, the larger caliber facilitates the collections. This could also be the reason for the best results encountered with the CMN program of the Spectra Optia blood cell separator. Since with this procedure, any single stop during the collection is a big deal, our nurse team tend to avoid using the CMN program if they do not have access through a very good central line. Therefore, these procedures are usually performed through CVC with the biggest caliber. As far as we know, this is the first time that these results are confirmed in a study.

Finally, we would like to highlight another relevant finding, which is the difference between the efficiency obtained with catheters placed in the upper part of the body and those placed in the femoral vein. This might be related to the size of those catheters, since there are clear differences among them when comparing each vein. On multivariate analysis this variable is no longer related to the efficiency, in contrast to the size of the CVC, which supports the idea that these variables are closely related. Physicians involved in CVC placement are likely to consider larger catheters whenever placing them in veins in the upper part of the body, which would also influence these results. Anyway, femoral CVCs obtained worse results in our study, so it would be advisable to place the CVC in the jugular vein whenever possible.

In conclusion and according to our results and others previously published, we consider that CVC-related complications in pediatric healthy donors are very rare, and therefore should specifically be considered in patients with serious illnesses. Furthermore, we must try to place catheters of the largest caliber possible without damaging the vessel, since the efficiency of the collection is significantly related to this variable.

## Data Availability

The raw data supporting the conclusions of this article will be made available by the authors, without undue reservation.
